# Supplementary material for: Area-time efficient pipelined number theoretic transform for CRYSTALS-Kyber
Source: PLoS One. 2025 May 14;20(5):e0323224. doi: 10.1371/journal.pone.0323224 (PMC12077723; doi:10.1371/journal.pone.0323224)
Supplement: Attachment — Submitted filename: Response to Reviewers.docx [file pone.0323224.s002.docx]

**Editor’s and Reviewer’s Comments**

The Authors would like to take this opportunity to appreciate Editor and Reviewers Constructive feedback and made sure that all the comments made by the worthy persons are addressed to the best of Author’s knowledge. Please find below the Authors response to the Editor/Reviewer comments. These are also being incorporated in the revised manuscript uploaded by the Authors.

| **Editor/Reviewers** | **Comments** | **Response** |
| --- | --- | --- |
| **Editor Comments** | 1.Please ensure that your manuscript meets PLOS ONE’s style requirements, including those for file naming. | The Authors have updated the manuscript to meet PLOS ONE’s style requirements. |
|  | 2. Please note that PLOS ONE has specific guidelines on code sharing for submissions in which author-generated code underpins the findings in the manuscript. In these cases, all author-generated code must be made available without restrictions upon publication of the work. | The Authors acknowledge PLOS ONE's code-sharing guidelines and confirm that the author-generated code will be made available without restrictions upon acceptance**.** |
|  | 3. Please update your submission to use the PLOS Latex template. | The Authors have revised the document to comply with PLOS ONE style requirements. The Authors acknowledge the journal’s requirement and ensure that submission in Latex template will be provided on acceptance. |
|  | 4. When completing data availability statement of the submission form, you indicated that you will make your data available on acceptance. We strongly recommend all authors decide on a data sharing plan before acceptance, as the process can be lengthy and hold up publication timelines. Please note that, though access restrictions are acceptable now, your entire data will need to be made freely accessible if your manuscript is accepted for publication. This policy adheres to all data except where public deposition would breach compliance with the protocol approved by your research ethics board. If you are unable to adhere to our open data policy, please kindly revise your statement to explain your reasoning and we will seek the editor’s input on an exemption. Please be assure that, once you have provided your new statement, the assessment of your exemption will not up hold the peer review process. | The Authors comply with PLOS ONE’s data-sharing requirements and confirm that we are aware that code sharing is mandatory. Upon acceptance, we will make the code freely accessible in a public repository, in full compliance with PLOS ONE’s conditions |
| **Reviewer # 1 Comments** | 1. The idea of MDC is not new, it was already used by Ref. 23 and this paper (10.1109/TC.2023.3296899). What is the novelty of the proposed MDC (Section 4.3 and 4.4) compared to these existing works? | A BRAM-free, iterative NTT implementation is proposed for CRYTSTALs-Kyber in Ref. 23. The NTT architecture is composed of a dual-butterfly that performs Forward NTT, Inverse NTT and Point-wise multiplication (PWM) operation.  MDC-based pipelined NTT architecture for CRYSTALs-Kyber is presented in paper (10.1109/TC.2023.3296899). The Radix-2 butterfly core in MDC chain performs NTT/INTT operation using resource sharing. Point-wise multiplication (PWM) is implemented using a separate hardware module.  The Authors have proposed MDC-based polynomial multiplication architecture which reconfigures the butterfly units to perform NTT, INTT, and PWM within a unified hardware unit, unlike the conventional MDC-based architectures.  In this study, two MDC-based architectures, a 2-parallel (MDC2NIP) and a 4-parallel (MDC4NIP) design, are presented along with elaborate discussion of execution of NTT, INTT, and PWM operations and data scheduling. Proposed architectures outperform in execution cycles when compared to NTT implementation in Ref. 23. Integrating the PWM operation within the MDC chain significantly reduces resource consumption of MDC2NIP and MDC4NIP in comparison with paper (10.1109/TC.2023.3296899) |
|  | 2. Section 4.1 is presenting many known results, they are not new and should not be considered as contributions/proposed ideas. | The Authors appreciate the Reviewer highlighting that section 4.1 presents already known optimizations. We acknowledge that Barrett reduction and LUT-based Karatsuba multiplication are not new techniques. However, the novelty of this work does not lie in the introduction of these individual components but rather in their carefully optimized integration within the proposed architecture.  The strategic selection is the result of an extensive literature review of various multiplication and reduction algorithms and analyzing their impact on resource utilization, clock frequency and computational complexity. The implementation of LUT-based Karatsuba multiplier with shift-add based Barrett reduction, eliminates the use of DSP, minimizing the resource consumption. The lightweight operations reduce the design complexity while maintaining a high computational throughput.  The above is also added in **section “Result Analysis”**, **page # 19**, **line # 622-628** along with a resource comparison table (Table 2) of our multiplier with different techniques presented in literature. |
|  | 3. The article is not written in a way easy to understand the contributions. The authors tend to report the detailed implementation techniques without making comparison with the SOTA. This is clearly shown in the contribution paragraph, the authors only mentioned some implementations that they have performed, without discussing why these are considered novel. Note that although the authors can achieve better area-time efficiency, it is also important to discuss why this happens. | The Authors appreciate the feedback from the Reviewer. The comparison of the adopted implementation techniques with SOTA and their novelty is added in the revised manuscript, **section “Proposed Architecture**”**, page # 5-6, line # 169-205**.  The performance results for adopting the optimized techniques in comparison with SOTA are added in the revised manuscript, **section “Result Analysis”, page # 19-20,** **line # 631-644.** |
|  | 4. It is advisable to share the source code so allow reproducible research. | The Authors acknowledge the Reviewer’s suggestion, and the code will be shared in a public repository on paper acceptance. |
| **Reviewer # 2 Comments** | 1. The title needs to be revised; usually, it does not contain a comma, so it is better to remove it after ‘efficient’. The word ‘parallel-pipelined’ does not make sense; it is better to use only pipelined, change ‘CRYSTALs’ to CRYSTALS. | As suggested by the Reviewer, changes are made in the **“Title”.** |
|  | 2. The abstract must include details of the implementation platform, such as the FPGA family/device and the associated tools. | The FPGA family/device along with the associated tools are added in the **“Abstract”**, **page #1, line #21-23**, as per comments of the Reviewer. |
|  | 3. How the functional verification of the design has been conducted, add details in the implementation section. | In accordance with the Reviewer’s suggestion, functional verification has been added in **section “Result Analysis”, page # 19,** **line # 613-617.** |
|  | 4. How the lookup table-based multiplier is efficient than the strategies adopted in Montgomery and interleaved multipliers presented in (1) point multiplication accelerator for arbitrary Montgomery curves, (2) efficient reconfigurable modular multipliers for post-quantum digital signatures, and (3) efficient soft core multipliers for digital signatures? | Lookup table-based multiplication is efficient as it precomputes partial results, reducing computational overhead and improving speed. The Authors will address each point of the Reviewer separately.  Point multiplication is a fundamental operation in Montgomery curves which is a special class of elliptic curves, used in Elliptic Curve Cryptography. The scheme involves computing multiples of a point on an elliptic curve expressed in the Montgomery form also known as point multiplication. A specialized hardware is designed consisting of modular multipliers to accelerate the operation on hardware. Montgomery Algorithm is well suited for computations for point multiplication in Montgomery curves as the numbers are in Montgomery form. Interleaved multipliers perform multiplication and reduction at the same time and reduce the intermediate storage in FPGA based designs. Hence, integration of interleaved multipliers based on Montgomery multiplication in point multiplication operations optimizes the computational efficiency  Using Lookup table-based multiplication method in point multiplication avoids full multiplications by storing precomputed values and thus reducing latency. The primary challenge is that large prime fields result in a larger memory overhead for storing precomputed results.  Montgomery and interleaved multipliers have been utilized in designing post-quantum digital signatures. Compared to Montgomery multiplication, which requires transformation into and out of Montgomery space and additional modular reductions, Lookup table based multiplication eliminates these extra steps, leading to lower latency.  A soft-core multiplier is a configurable hardware multiplier implemented using lookup tables (LUTs), flip-flops (FFs) and shift registers instead of dedicated hardwired multipliers (DSP blocks). A soft-core multiplier consists of a partial product generator, adder tree and reduction logic. Lookup table multipliers are a type of soft-core multipliers that store the precomputed multiplication in LUTs. This multiplier architecture results in lower latency, fewer computational resources, less power consumption and faster modular multiplication when compared to Montgomery and interleaved multipliers. |
|  | 5. It is demonstrated in Table 3 that the proposed butterfly with FIFO consumes around 1302, 420 LUTs in MDC2NIP, and even it jumps to 2604, 840 in case of MDC4NIP. How the overall design can consume just 1884 LUTs. Clarify how many BU you are using in the MDC2 and MDC4. Add details of your BU, such as LUTs, FFs, and clock cycle consumption. Moreover, how are you handling div by 2 in GSBU? | MDC2NIP consists of seven butterfly units (BUs). Table 4 in the revised manuscript, presents the resources of all the butterfly units in MDC2NIP that is 1302 LUTs and 420 FFs. MDC4NIP consists of fourteen butterfly units and hence the BUs occupy twice the resources than in MDC2NIP, that is 2604 LUTs and 840 FFs.  In MDC2NIP design, 7 BUs and 6 commutators occupy 1302 and 125 LUTs. Distributed LUT-based BROM for twiddle factor storage (TWROM), FIFO in MDC chain [FIFO(MDC)] and FIFO for storing input/output coefficients of NTT, INTT and PWM operation [FIFO(Memory)] consumes 74, 95 and 288 LUTs. This equals a total of 1884 LUTs.  In MDC4NIP architecture, 14 BUs, 12 commutators, TWROM, FIFO (MDC) and FIFO (Memory) occupy 2604, 250, 74, 193 and 288 LUTs, which results in a total of 3409 LUTs.  This is also mentioned under the section of **“Result Analysis”, on Page # 22, Line # 723-732.**  The INTT operation using Gentlemen Sande Butterfly unit (GSBU) requires a Div 2 operation. In this work we have incorporated this by dividing the pre-computed twiddle factors *w* by 2 and stored *w*/2 in LUT- based distributed ROM.  This is mentioned in **Section “Proposed Archiecture”, subsection “Butterfly unit”, page # 9 and line # 285-288.** |
|  | 6. As the design is BRAM free, how many twiddle factors do you need to store in NTT and INTT, and how many LUTs/FFs are being consumed for just this storage? | In this work, we have designed custom LUT-based distributed memories to store twiddle factors (TWs) for all butterfly units in MDC2NIP and MDC4NIP. The NTT, INTT and PWM computation require 384 TWs (128+128+128) in Kyber.  In 7 series FPGA, 6 input LUTS are used to store up to 2^6^=64 bits. This implies we can implement a 64x1 bit ROM using one LUT. Each stage in MDC2NTT and MDC4NTT has a different requirement of TWs and we have designed a custom-built ROM in accordance to it. For example, the first stage in MDC2NTT, when working in NTT, INTT and PWM mode needs 1, 64, 0 TWs. The total number of twiddle factors for this stage is 65 and the width of each coefficient is 12-bits, which are stored in a 65x12 bit ROM and uses 13 LUTS. The second stage requires 2, 32, 128 TW for NTT, INTT and PWM operations. Total number of TWs is 162 and is implemented using 31 LUTs. The TWs for remaining stages occupy 4,3,4,6,13 respectively resulting in a total of 74 LUTs.  MDC4NIP requires the same number of TWs for each stage as in MDC2NIP. The total number of TWs of each stage in MDC2NIP is divided into two LRMs in MDC4NIP, this helps in data to be processed independently and concurrently in the two MDC-chains.  The details of Twiddle factor storage and management has been mentioned in **section** **“Proposed Archiecture”, subsection “TW management” on Page # 18-19, Line # 600-609.** |
|  | 7. In Kyber, how many PWM are required, and in your setting, how many clock cycles are consumed in its computation? | In CRYSTALS-Kyber, matrix-vector and vector-vector multiplications are performed using NTT-based polynomial multiplication. The number of PWM operations required in Kyber depends on the parameter k, which varies for different security levels. Different values 2,3,4 of k correspond to Kyber-512, Kyber-768 and Kyber-1024.  This paper presents polynomial multiplication of two polynomials during vector-vector or matrix-vector multiplication in Kyber. Each polynomial has 256 coefficients of 12-bit each. For multiplication the polynomials are first transformed into NTT domain using forward NTT.  The NTT-transformation results in two 1-degree polynomials and one PWM operation consists of five multiplication and two addition operations, resulting in two coefficients per operation. After 128 PWM operations, 256 output coefficients are then transformed back into the original domain by applying an inverse NTT-transform.  In proposed MDC2NIP, a total of 262 cycles are required to perform PWM operation, where 6 cycles are pipeline delay of MDC chain in PWM mode and 256 cycles are required to compute 1 coefficients per cycle.  This is mentioned in revised manuscript, **section “Proposed Architecture”, subsection, “MDC2PWM”, page #13 line #416-422.**  A total of 134 cycles are required to perform PWM operation in MDC4NIP, where 6 cycles are pipeline delay of MDC chain in PWM mode and 128 cycles are required to compute 2 coefficients per cycle.  This is mentioned in revised manuscript, **section “Proposed Architecture”, subsection, “MDC4PWM”, page #17, line #541-546.** |
|  | 8. Some recent work must be added and compared in Table 2, such as (1) A better Kyber Butterfly for FPGAs, (2) Efficient Number Theoretic Transform Architecture for CRYSTALS-Kyber, etc. | The Authors appreciate the Reviewer’s suggestion and a recent work of NTT-based polynomial multiplier for CRYSTTALs-Kyber, performing NTT, INTT and PWM operation is added in the comparison table **“Table 3”** **(15^th^ row, Ref 23),** **section “Result Analysis” page #20.**  The analysis of ATP result of Author’s architectures with Ref. 23, is given in **section “Result Analysis”, Page #22, Line #695-697.**  A butterfly unit is the main processing element incorporated in NTT/INTT operation. As Table 3 only compares the state of the architectures performing complete NTT, INTT and PWM operations, the Authors did not include a butterfly implementation in the comparison table.  However, if the Reviewer requires a separate comparison focusing specifically on the butterfly architecture, the Authors would be happy to provide it as an additional comparison. |
|  | 9. Most of the schemes are now hybrid, so how will your design scale in a multi-scheme environment where lattice and hash-based schemes are integrated? | The Authors acknowledge the Reviewer’s insights regarding the scalability of our design in a multi-scheme environment. The CRYSTALs-Kyber and CRYSTALs-Dilithium have different parameters of prime modulus q, 3329 and 8380417, respectively. The condition of scalability can be achieved by designing a unified butterfly architecture that supports both q=3329 and 8380417. By designing unified butterfly units, the proposed MDC2NIP and MDC4NIP architectures can efficiently perform polynomial multiplication for both Kyber and Dilithium schemes.  While this paper focuses on Kyber-specific implementation, further optimizations and hybrid implementations have been identified as future research directions and added in **section** **“Future work”, on Page # 23, Line # 756-758.** |
|  | 10. Add throughput per slice figure in Table 2 and compare your design with the listed designs on this factor as well. | Throughput (TP) is calculated as (no. of bits x maximum clock frequency)/Latency in clock cycles. This value is then divided by total number of slices to compute throughput per slice (TP/slice).  The proposed architecture and the others listed in the comparison table have reported different cycles for NTT, INTT and PWM operation. As the number of execution cycles are different the throughput for each operation will also be different.  As per the reviewer's comments we have added the throughput and throughput per slice statistics for NTT operation in Table 3.  It is important to note that TP and TP/slice are not reported in works listed in the comparison table and have been calculated by the Authors based on the reported clock frequency, latency (clock cycles) and the number of bits as input per cycle in the designs.  The results indicate that our proposed MDC4NIP achieves the best TP/A, providing a clearer evaluation of our resource efficiency. The analysis of comparison is given in **section “Result Analysis”, page # 22, Line # 706-722**. |
|  | 11. Power consumption is very important, add power consumption stats and compare it with the listed designs. | The Authors appreciate the Reviewer’s insightful comments and acknowledge the analysis of power consumption for optimizing FPGA-based designs.  An area-time efficient design contributes to low power consumption, as FPGA power consumption is largely dependent on logic utilization and switching activity. In this study, we have focused on performance improvements by reducing area utilization and improving execution cycles. Proposed designs achieve significant resource efficiency, indicating a power efficient design. However, we have not explicitly analyzed power consumption as part of our evaluation.  A detailed power analysis will be incorporated in our future research and is mentioned in **section** “**Future Work**”, **on Page # 23, Line # 758-760s.**  . |
|  | 12. Side-channel attacks are very important nowadays. How robust is your design against these attacks (power and timing)? | Power Analysis Attacks rely on variations in power consumption when processing secret-dependent operations. Various techniques can be implemented in designs to mitigate power analysis attacks such as constant power consumption, noise injection, blinding techniques and masked arithmetic operations. Timing attacks can be prevented by constant time execution, branch elimination and instruction scheduling equalization.  Our design is implemented to run in constant time and maintain constant power consumption, ensuring resilience against timing and power-based side-channel attacks. However, a comprehensive side-channel analysis has not yet been conducted. This remains an important aspect of future work to evaluate and further enhance the security of the proposed architecture against advanced side-channel attacks. This has been identified **in section “Future Work**”, **on Page # 23, Line # 758-760.** |
