# Supplementary material for: Area-time efficient pipelined number theoretic transform for CRYSTALS-Kyber
Source: PLoS One. 2025 May 14;20(5):e0323224. doi: 10.1371/journal.pone.0323224 (PMC12077723; doi:10.1371/journal.pone.0323224)
Supplement: Attachment — Submitted filename: Response_to_Reviewers_auresp_2.docx [file pone.0323224.s003.docx]

**Editor’s and Reviewer’s Comments**

The Authors would like to take this opportunity to appreciate Editor and Reviewers constructive feedback and made sure that all the comments made by the worthy persons are addressed to the best of Author’s knowledge. Please find below the Authors response to the Editor/Reviewer comments. These are also being incorporated in the revised manuscript uploaded by the Authors.

| **Editor/Reviewers** | **Comments** | **Response** |
| --- | --- | --- |
| **Journal Requirements** | Please review your reference list to ensure that it is complete and correct. If you have cited papers that have been retracted, please include the rationale for doing so in the manuscript text, or remove these references and replace them with relevant current references. Any changes to the reference list should be mentioned in the rebuttal letter that accompanies your revised manuscript. If you need to cite a retracted article, indicate the article’s retracted status in the References list and also include a citation and full reference for the retraction notice. | To the best of Author’s knowledge, no retracted papers are present in the reference list. |
| **Editor Comments** | Please address the remaining points from the previous review in the manuscript**,** not just as rebuttals. | As per the Editor’s comments, the remaining points from the rebuttal of previous review have been added in the revised manuscript.  The Author’s response to comment #1 of Reviewer #1, has been added in **section** **“Proposed Architecture”, on Page #5-6, Line #168-181.** |
|  | Also, consider sharing, fully or partially, your codes to encourage reproducible research in the community. | The Authors acknowledge the PLOS one policy for code sharing, and the code will be shared on acceptance. |
| **Reviewer # 2 Comments** | 1. The authors provided details in the response letter for the concern " How the lookup table-based multiplier is efficient than the strategies adopted in Montgomery and interleaved multipliers presented in (1) point multiplication accelerator for arbitrary Montgomery curves, (2) efficient reconfigurable modular multipliers for post-quantum digital signatures, and (3) efficient soft core multipliers for digital signatures?". But this discussion is not reflected in any part of the revised manuscript. It is important to add this because this will add another dimension that reduction can be done using interleaved fashion. | The Author’s appreciate the Reviewer’s feedback, and the suggested discussion has been added in **section** **“Proposed Architecture”, subsection, “Modular Multiplication” on Page #7-8, Line #229-254.** |
|  | 2. The Response against another concern " Some recent work must be added and compared in Table 2, such as (1) A better Kyber Butterfly for FPGAs, (2) Efficient Number Theoretic Transform Architecture for CRYSTALS-Kyber, etc." is not satisfactory. The better Kyber paper also reported a complete NTT/INTT implementation results. Both these manuscript are recent proposals so must be compared. | As per the Reviewer’s comments, the Authors have added the suggested papers in Table 3 for comparison with the proposed work.  Paper (1), added as Ref. 48 in revised manuscript, presents a novel design for modular reduction. The compact design is used to implement a butterfly unit for CRYSTALS-Kyber. To evaluate the efficiency of the proposed design, the authors in Ref. 48, replaced the butterfly unit in an existing open-source NTT-based polynomial multiplication (Ref. 14) for Kyber, with their own optimized butterfly unit. This allowed for a direct comparison of area utilization, demonstrating a significant reduction in LUT usage. While preliminary findings indicate that the latency of the design in paper (Ref. 48) is lower than that of the original open-source implementation (Ref. 14), the clock frequency and number of clock cycles are not reported in Ref. 48. Therefore, the comparison of our proposed MDC2NIP and MDC4NIP with Ref. 48 is limited to area resource utilization only. The three implemented designs in Ref.48 are added in **“Table 3”** **(Row #19-21),** **section “Result Analysis” page #21.**  The above along with the resource comparison is given in **section “Result Analysis”, Page #23, Line #744-754.**  Paper (2), added as Ref. 47 in revised manuscript, proposes a unified Butterfly Unit (UBU) that combines interleaved multiplication, radix-4 processing, and resource-sharing techniques to perform NTT and inverse NTT (INTT) operations efficiently. The work implements three NTT/INTT architectures lightweight, balanced and low latency designs. The three implemented designs in Ref.47 are added in **“Table 3”** **(Row #16-18),** **section “Result Analysis” page #21.** As PWM operation is not implemented in Ref.47, clock cycles, execution time and ATP values for PWM have not been mentioned in Table 3.  The above along with the ATP comparison for NTT/INTT operation is given in **section “Result Analysis”, Page #23, Line #737-743.** The throughput comparison is also provided in **section “Result Analysis”, Page #24, Line #775-780.** |
|  | 3. Power consumption figures are missing again. Vivado provides a simple way to measure the power so it is better to add power consumption figures. | The Authors appreciate the Reviewer’s valuable comments, and the power figures have been added in **section** **“Result analysis”, on Page #24, Line #798-799.** |
